# Health care seeking patterns and determinants of out-of-pocket expenditure for Malaria for the children under-five in Uganda

**DOI:** 10.1186/1475-2875-12-175

**Published:** 2013-05-31

**Authors:** Juliet Nabyonga Orem, Frederick Mugisha, Albert Peter Okui, Laurent Musango, Joses Muthuri Kirigia

**Affiliations:** 1 Health Systems and Services Cluster, WHO Uganda Office, 24578, Kampala, Uganda; 2 Department of Economic Development Policy and Research, Ministry of Finance, Planning and Economic Development, Kampala, Uganda; 3 Malaria Control Programme, Ministry of Health, Kampala, Uganda; 4 Health Systems and Services Cluster, WHO Regional Office for Africa, Brazzaville, Congo

**Keywords:** Household out-of-pocket expenditure, Uganda, Under-fives, Malaria

## Abstract

**Background:**

The objectives of this study were to assess the patterns of treatment seeking behaviour for children under five with malaria; and to examine the statistical relationship between out-of-pocket expenditure (OOP) on malaria treatment for under-fives and source of treatment, place of residence, education and wealth characteristics of Uganda households. OOP expenditure on health care is now a development concern due to its negative effect on households’ ability to finance consumption of other basic needs.

**Methods:**

The 2009 Uganda Malaria Indicator Survey was the source of data on treatment seeking behaviour for under-five children with malaria, and patterns and levels of OOP expenditure for malaria treatment. Binomial logit and Log-lin regression models were estimated. In logit model the dependent variable was a dummy (1=incurred some OOP, 0=none incurred) and independent variables were wealth quintiles, rural versus urban, place of treatment, education level, sub-region, and normal duty disruption. The dependent variable in Log-lin model was natural logarithm of OOP and the independent variables were the same as mentioned above.

**Results:**

Five key descriptive analysis findings emerge. First, malaria is quite prevalent at 44.7% among children below the age of five. Second, a significant proportion seeks treatment (81.8%). Third, private providers are the preferred option for the under-fives for the treatment of malaria. Fourth, the majority pay about 70.9% for either consultation, medicines, transport or hospitalization but the biggest percent of those who pay, do so for medicines (54.0%). Fifth, hospitalization is the most expensive at an average expenditure of US$7.6 per child, even though only 2.9% of those that seek treatment are hospitalized.

The binomial logit model slope coefficients for the variables richest wealth quintile, Private facility as first source of treatment, and sub-regions Central 2, East central, Mid-eastern, Mid-western, and Normal duties disrupted were positive and statistically significant at 99% level of confidence. On the other hand, the Log-lin model slope coefficients for Traditional healer, Sought treatment from one source, Primary educational level, North East, Mid Northern and West Nile variables had a negative sign and were statistically significant at 95% level of confidence.

**Conclusion:**

The fact that OOP expenditure is still prevalent and private provider is the preferred choice, increasing public provision may not be the sole answer. Plans to improve malaria treatment should explicitly incorporate efforts to protect households from high OOP expenditures. This calls for provision of subsidies to enable the private sector to reduce prices, regulation of prices of malaria medicines, and reduction/removal of import duties on such medicines.

## Background

The objectives of this study were to assess the patterns of treatment seeking behaviour for children under five with malaria; and to examine the statistical relationship between out-of-pocket expenditure (OOP) on malaria treatment for under-fives and source of treatment, place of residence, education and wealth characteristics of Uganda households. Household OOP expenditure on health is now a development concern due to its negative effect on households’ ability to finance consumption of other basic needs and invest in human capital [1,2]. In countries without prepayment mechanisms, OOP expenditure on health can impoverish households subsequently impacting the entire economy [1].

The direct and indirect effects of malaria have been shown to have a negative significant impact on economic growth [3,4]. The mortality and morbidity due to malaria is significant, with up to 30% of out-patients’ attendances in health facilities in Uganda being due to malaria [5]. Children under five years of age are disproportionately affected. For example, it is estimated that in Uganda malaria-specific mortality is between 70,000 and 100,000 child deaths per year [6].

### The Ugandan health system

The health care delivery system in Uganda is organized into five tiers and the package of health services to be provided at the different levels has been detailed [7]. National referral hospitals provide highly specialized services, research and training; regional referral hospitals provide a limited package of specialized services, teaching, research and supervision of clinical services in their areas of jurisdiction. General hospitals provide inpatient, surgical and outpatient services; health centre IV provide emergency surgical services, inpatient and outpatient services while lower level health centres II and III provide mainly ambulatory care and outreach services. The health system is pluralistic comprised of public, private not for profit, private for profit and traditional/complementary medicine practitioners.

Malaria treatment in Uganda is administered through public hospitals and health centres, private not-for-profit hospitals and health centres and, private for profit outlets which encompasses hospitals, clinics and drug shops. Service provision in the public sector is free and in addition, the Ministry of Health has put in place village health teams (VHT) comprised of five community-owned resource persons looking after 10 households on average. VHTs are trained to conduct health education and provide treatment for minor ailments to children below five years, including treatment of malaria [8].

The health system is financed from multiple sources including public (central and local governments), donors, household OOP, NGOs and employers. Total health expenditure (THE) per capita was estimated at US$52 in 2009/10 financial year of which 36% was from donors and global health initiatives, 16% from public sources and 17% from non-governmental organizations and employers. Household OOP is the predominant source accounting for 42% THE [9].

Malaria policy in Uganda has not focused specifically on addressing household OOP expenditure [10]. The extent to which OOP expenditure deters early diagnosis and treatment not only undermines malaria control efforts, it also affects households’ participation in the labour market in the short and long term [11]. Furthermore, it influences health seeking behaviour subsequently impacting on malaria treatment outcomes. The analysis in this paper provides lessons for malaria control programmes, which need to be addressed in order to ensure attainment of malaria control objectives.

## Methods

### Data sources

The 2009 Uganda Malaria Indicator Survey was the source of data for this analysis [12]. The main objective of the survey was to determine the progress made in the prevention and control of malaria. The field work for the survey was conducted in the months of November and December 2009 from a nationally representative sample of 4,760 households in 170 census enumeration areas. All women age 15–49 years in these households were eligible for individual interviews, during which they were asked questions about malaria prevention during pregnancy and treatment of childhood fevers. In addition, the survey included testing for anaemia and malaria among children age 0–59 months using finger (or heel) prick blood samples. Only data on children regarding health-seeking patterns and OOP expenditure – that is, amount spent in the process of a child getting treatment - was used in this analysis [13].

The Uganda Bureau of Statistics and the Uganda Malaria Surveillance Project conducted the survey on behalf of the National Malaria Control Programme of the Ministry of Health. The survey was designed to provide national, regional, urban, and rural estimates of key malaria indicators stratified into nine survey regions plus the capital city Kampala (see Table 1). Each of the nine regions consisted of eight to ten contiguous administrative districts of Uganda.

**Table 1:** Regions and districts covered

**Region**	**District**
Central 1	Kalangala, Masaka, Mpigi, Rakai, Lyantonde, Sembabule, and Wakiso
Central 2	Kayunga, Kiboga, Luwero, Nakaseke, Mubende, Mityana, Mukono, and Nakasongola
Kampala	Kampala
East Central	Jinja, Iganga, Namutumba, Kamuli, Kaliro, Bugiri, and Mayuge
Mid-eastern	Kapchorwa, Bukwa, Mbale, Bududa, Manafwa, Tororo, Butaleja, Sironko, Pallisa, Budaka, and Busia
North-east	Kotido, Abim, Kaabong, Moroto, Nakapiripirit, Katakwi, Amuria, Bukedea, Soroti, Kumi, and Kaberamaido
Mid-northern	Gulu, Amuru, Kitgum, Pader, Apac, Oyam, Lira, Amolatar, and Dokolo
West Nile	Moyo, Adjumani, Yumbe, Arua, Koboko, Nyadri, and Nebbi
Mid-western	Masindi, Buliisa, Hoima, Kibaale, Bundibugyo, Kabarole, Kasese, Kyenjojo, and Kamwenge
South-western	Bushenyi, Rukungiri, Kanungu, Kabale, Kisoro, Mbarara, Ibanda, Isingiro, Kiruhura, and Ntungamo

### Descriptive statistics

For each child under five years old, questions were asked on: whether the child was ill with a fever in the last two weeks (yes, no); and whether they sought treatment and where. Table 2 shows selected descriptive statistics without sample weights mainly for consistency checks.

**Table 2:** Selected descriptive statistics

	**Number**	**%**
Child was ill with a fever in the last 2 weeks?		
No	2,092	55.8
Yes	1,657	44.2
Total	3,749	100
Did child seek treatment from anywhere?		
No	294	17.74
Yes	1,363	82.26
	1,657	100.0
Place first sought treatment for fever?		
Public	686	50.33
Private	660	48.42
Traditional	15	1.1
Other	2	0.15
Total	1,363	100

### Analysis frame

To explore OOP expenditure for malaria among children below five years of age, simple proportion and averages were compared as an initial step. These include the proportion of children who reported fever, those who sought treatment, the place where they first sought treatment for fever, the proportion that incurred OOP expenditure, and the average OOP expenditure. Components of OOP included expenditures on consultation, medicine, transport and hospitalization.

Results are reported separately according to urban or rural, region, mother’s education and wealth quintiles. However, because observed changes could be related to other factors, binomial logit and Log-lin models were estimated to control for these factors. The dependent variable in logit model is a dummy variable, which takes a value of one if a child incurred OOP; and zero otherwise. The dependent variable in the Log-lin model is the natural log of OOP [14].

The Log-lin model was chosen for two reasons. One, the Log-lin models are frequently used in economics since consumption functions tend to increase at a decreasing rate past some level of income (or wealth) [14]. Engel curves are used to explain the relationship between consumer expenditure on a particular good (in our case malaria treatment services) and his/her income (in our case wealth), holding other factors constant. Use of semi-log functional form, such as log-lin, is justified whenever the item’s consumption can be expected to tail off as income (or wealth) increases. Two, the natural log of OOP (dependent variable) is used as it normalizes the OOP expenditure, and it was deemed the best fit based on skewedness/kurtosis tests for normality and probability plot.

In a log-lin model, the slope coefficient *β*_1_ measures the relative change in OOP for a given absolute change in X, i.e. *β*_1_ = *relative change in OOP*/*absolute change in X*. In a Log-lin model if a specific independent variable *X*_1_ changes by one unit, then OOP will change by *β* times 100 percent, holding other factors constant. Thus, the estimated slope coefficient after multiplying by 100 measures the rate of growth in OOP.

In both models, the independent variables are the same. They include: wealth quintiles (with the poorest being the base and the richest expected to have a positive sign); rural *vs* urban (with the urban as a base and rural expected to have a negative sign); place of treatment (with public as the base and the private expected to have a positive sign); education level (with no education as the base and higher education expected to have a positive sign); sub-region (with central 1 as the base and no expected pattern for other sub-regions, except for Kampala with a positive sign); and normal duty disruption (with no disruption as the base and disruption expected to have a positive sign).

In the binomial logit model, the standardized normal distribution (z) is used to test whether an estimated slope coefficient is significantly different from zero, i.e. whether *H*_0_: *β*=0 or *H*_*A*_: *β*≠0. The null hypothesis (*H*_0_: *β*=0) is rejected and alternative hypothesis (*H*_*A*_: *β* ≠ 0) accepted if the computed value of the z-distribution (z_*c*_) is greater than critical value of the z-distribution (z_*a*_) at 5% level of significance. For example, for a 5% two-tailed test z_*a*_=1.96 so any z_*k*_ larger than 1.96 would lead to rejection of *H*_0_ and declaration that *β*_*k*_ is significantly different from zero at the 95 per cent level of confidence. At 1% level of significance the z_*a*_=2.58_._

In the Log-lin model, the t-distribution is used to test whether an estimated slope coefficient is significantly different from zero, i.e. whether *H*_0_: *β*=0 or *H*_*A*_: *β*≠0_._ The null hypothesis *H*_0_: *β*=0 is rejected and alternative hypothesis (*H*_*A*_: *β*≠0) accepted if the computed value of the t-distribution (*t*_*c*_) is greater than critical value of the t-distribution (*t*_*a*_) at 5% level of significance. For example, for a 5% two-sided test with 913 degrees of freedom, *t*_*c*_=1.96 so any *t*_*k*_ larger than 1.96 would lead to rejection of *H*_0_ and declaration that *β*_*k*_ is significantly different from zero at the 95 percent level of confidence. At 1% level of significance the *t*_*c*_=2.576.

The binomial logit and Log-lin models were estimated using the STATA computer software.

### Limitations of the study

First, the analysis reported in this paper is based on Uganda Malaria Indicator Survey that was conducted to determine the progress made in the prevention and control of malaria. It was not dedicated to the purpose of assessing the patterns of treatment seeking behaviour for children under five with malaria; and exploring the relationship between OOP on malaria treatment for under-fives and explanatory variables reported in this paper. Therefore, it is not possible to know whether there might have been data collection errors lurking in the dataset. Second, questions on household real income were not included in the survey, and thus, wealth had to be used as a proxy. Third, regression diagnostic tests were not performed to ensure that the binomial logit and Log-lin models were the most appropriate.

## Results

The results of the analysis are arranged in three parts. The first part examines which children had fever and the ones that sought health care and from which provider. The second part takes a closer look at those who sought care and attempts to examine which children are likely to incur OOP expenditure. The third part examines the patterns and levels of OOP expenditure. In all cases, tables are used to present a spectrum of results.

### Fever and health care seeking behaviour

There were no major variations in the percentage of children with fever in the two weeks preceding the survey across urban and rural, among the poor and the rich, and across the various levels of educational attainment (Table 3). What differs is in the sub-regions: Kampala and south-western sub-regions reported the lowest at about 22% while the mid-northern reported the highest at 66.1%.

**Table 3:** Children under five years with fever and those that sought advice or treatment in Uganda

**Background characteristics**	**% with fever**	**#**	**% seek advice or treatment**	**% seek from one source**	**Place first sought treatment**				
					**Public**	**Private**	**Traditional**	**Other**	**Total**
Rural - Urban									
Urban	47.8	396	68.5	97.2	26.8	73.2	0.0	0.0	100
Rural	44.3	3,353	83.9	92.0	46.8	51.7	1.5	0.1	100
Sub-region									
Central 1	33.9	319	85.2	95.1	41.6	58.4	0.0	0.0	100
Central 2	54.3	332	83.4	91.0	37.6	59.4	3.0	0.0	100
Kampala	22.4	211	87.2	100.0	43.0	57.0	0.0	0.0	100
East Central	56.3	468	60.1	95.3	33.0	66.9	0.2	0.0	100
Mid-eastern	30.1	402	83.5	87.9	43.6	47.0	9.1	0.3	100
North-east	52.4	415	90.6	92.3	45.7	54.4	0.0	0.0	100
Mid-northern	66.1	422	91.2	91.1	47.7	52.2	0.0	0.1	100
West Nile	51.8	461	81.4	89.5	46.6	50.6	2.8	0.0	100
Mid-western	36.4	407	77.7	94.3	59.9	40.1	0.0	0.0	100
South-western	22.3	312	96.9	98.3	47.6	52.5	0.0	0.0	100
Mother's education level									
No education	46.1	861	79.7	91.7	56.3	42.7	1.0	0.0	100
Primary	45.8	2,281	82.1	92.6	43.0	55.5	1.5	0.1	100
Secondary	40.0	541	81.3	94.3	36.0	62.7	1.0	0.2	100
Higher	32.7	66	95.4	90.2	18.4	80.6	1.0	0.0	100
Wealth index									
Poorest	55.1	971	82.9	91.5	51.4	47.6	1.1	0.0	100
Poorer	45.2	727	85.4	92.7	42.4	54.5	3.0	0.1	100
Middle	44.0	730	75.7	91.0	47.9	51.2	0.7	0.2	100
Richer	40.4	695	87.6	91.7	37.6	62.3	0.1	0.0	100
Richest	35.9	626	75.7	98.6	37.1	61.6	1.4	0.0	100
Total	44.7	3,749	81.8	92.6	44.4	54.2	1.3	0.1	100

# is number of children.

Differences are not large, even though they follow the expected pattern, regarding whether they sought treatment for children with fever in the two weeks preceding the survey. There is however one notable exception: a higher percentage of children in rural areas sought treatment than in urban areas. This is the same pattern observed for the richest and poorest 20%. A higher percentage of children in the poorest 20% compared to those in the richest 20% households sought treatment. It is not clear why this is the case because the level of education attainment follows the expected pattern.

What the results also show is that the majority of patients (92.6% nationally) seek health care from one source as opposed to multiple sources. The pattern is the same across the various categories: sub-region, urban *versus* rural, educational attainment and wealth index. Because of this observation, analysis of the first place of treatment gives a general understanding of patient choice. In the last five columns of Table 3 the percentage of children that received treatment at public, private, and traditional is shown. A category of others is added to cater for the unknown, missing and those that could not be classified as either public, private or traditional. The last column is an indication that the previous four columns add up to 100%.

The results show an important issue in regard to patient choice between public and private providers. With the exception of the poorest, those children whose mothers have no education, and the children in mid-western, the patient choice is the private provider. Preference for a private provider is more pronounced in urban, in those whose parents have at least secondary education, and in the richest 40% of the wealth index. Drug shops are part of the private sector and given their penetration within the country, they are easily accessible.

### Likelihood of out-of-pocket expenditure

#### Binary comparisons

Results show that more than half (50%) of those that sought health care did incur OOP expenditure (Table 4). So the simple point is that one has to have some money at hand to seek health care.

**Table 4:** Percentage of children that sought advice or treatment that paid out-of-pocket the different components by population categories

**Background characteristics**	**Any**	**Consultation**	**Medicine**	**Transport**	**Hospitalization**
Urban	82.2	47.9	56.1	27.1	1.8
Rural	69.4	23.1	53.7	13.6	3.0
Sub-region					
Central 1	63.1	13.7	51.6	17.0	0.8
Central 2	80.9	19.9	57.4	28.3	3.1
Kampala	89.4	27.3	59.0	30.9	5.0
East central	73.6	51.5	55.7	17.5	1.4
Mid-eastern	81.6	37.4	69.5	19.0	6.3
North-east	62.8	8.0	58.0	2.4	0.3
Mid- northern	68.4	21.9	48.6	9.3	3.0
West Nile	68.0	14.5	57.1	8.5	4.9
Mid-western	66.1	26.2	50.6	13.3	3.8
South-western	71.4	37.7	41.1	33.1	3.1
Mother's education level					
No education	64.4	25.8	46.7	13.5	5.5
Primary	69.7	21.6	53.4	13.0	2.2
Secondary	84.0	42.3	66.4	23.8	2.5
Higher	94.3	65.4	73.5	45.4	0.0
Wealth index					
Poorest	59.7	12.5	49.7	8.5	3.2
Poorer	74.9	27.8	58.2	13.8	3.4
Middle	72.3	27.4	49.3	17.4	2.9
Richer	73.5	34.5	55.5	15.2	2.6
Richest	82.5	38.3	60.7	28.5	1.7
Total	70.9	26.0	54.0	15.2	2.9

There are however significant differences: those in urban areas are more likely to incur OOP expenditure than those in rural areas; those in the richest 20% households are more likely to incur OOP expenditure than those in the poorest 20%; those whose mothers have a higher level of education are more likely to incur OOP expenditure than those whose mothers have a lower level of education. In terms of regions, those who are less likely to incur OOP expenditure are in the Karamoja region, which includes the districts of Kotido, Abim, Kaabong, Moroto, Nakapiripirit, Katakwi, Amuria, Bukedea, Soroti, Kumi, and Kaberamaido. Districts that are more likely to incur OOP, with the exception of Kampala, are Kapchorwa, Bukwa, Mbale, Bududa, Manafwa, Tororo, Butaleja, Sironko, Pallisa, Budaka, and Busia.

Among those that sought health care, a higher percentage incurred OOP expenditure on medicine (54.0%) as opposed to consultation (26.0%), transport (15.2%) and hospitalization (2.9%) nationally (Table 4). The picture is similar across the various categories. However, consultation seems to be a preserve of children whose mother’s education is at least high school.

#### Multivariate comparison

The binomial logit regression results are reported in Table 5. The slope coefficients, odds ratio (odds ratio equals exponential of coefficient) and computed z values are reported. It is indicated for each of the variables whether they are statistically significant at 5% or 1% shown by * and ** respectively. Thus, whenever the computed z value is larger than the critical z value of 1.96, the null hypothesis that a slope coefficient is equal to zero is rejected at 95 per cent level of confidence. Similarly, when the computed z value is larger than the critical z value of 2.58, the null hypothesis is rejected at 99% level of confidence.

**Table 5:** Binomial logit model estimates of paying out-of-pocket for children under-five with malaria, for different categories of the population in Uganda

**Paid out of pocket**	**Coef**	**Odds ratio**	**Std Err**	**z**	**P>z**	
Wealth Index(Poorest)						
Poorer	0.42	1.5	0.20	2.05	0.040	*
Middle	0.41	1.5	0.22	1.88	0.060	
Richer	0.43	1.5	0.24	1.77	0.076	
Richest	0.95	2.6	0.31	3.02	0.003	*
Rural	−0.14	0.9	0.37	−0.37	0.714	
Place first sought treatment						
(public facility)^$^						
Private	2.60	13.4	0.17	15.53	0.000	**
Don’t know	0.23	1.3	0.66	0.36	0.721	
Sought treatment from one source	−2.08	0.1	0.31	−6.78	0.000	**
Highest educational level						
(no education)						
Primary	−0.02	1.0	0.17	−0.1	0.921	
Secondary	0.51	1.7	0.28	1.83	0.068	
Higher	1.46	4.3	1.16	1.26	0.207	
Sub-region(Central 1)						
Central 2	1.24	3.4	0.35	3.56	0.000	**
Kampala	1.44	4.2	0.69	2.09	0.036	*
East central	1.28	3.6	0.35	3.63	0.000	**
Mid-eastern	1.31	3.7	0.38	3.46	0.001	**
North-east	0.69	2.0	0.34	2.05	0.040	*
Mid-northern	0.82	2.3	0.33	2.47	0.013	*
West Nile	0.67	2.0	0.33	2.03	0.043	*
Mid-western	1.35	3.9	0.36	3.73	0.000	**
South-western	1.00	2.7	0.43	2.31	0.021	*
Normal duties disrupted	0.78	2.2	0.16	4.79	0.000	**
Constant	0.44	1.6	0.87	0.51	0.610	
Number of observations					1,357	
Pseudo R2					0.2645	
LR chi2(21)					444.63	

^$^Traditional healer omitted due to small numbers.

** Statistically significant at 1% level.

*Statistically significant at 5% level.

In the binomial logit model, the slope coefficients for the variables Richest wealth quintile, Private facility as first source of treatment and sub-regions Central 2, East central, Mid-eastern, Mid-western, and Normal duties disrupted were positive and statistically significant at 99% level of confidence (z_*c*_>z_*a*_). Similarly, the slope coefficients for variables Poorer wealth index and sub-regions Kampala, North-east, Mid-northern, West Nile, and South-Western were positive and statistically significant at 95% level of confidence. On the contrary, the slope coefficient for the variable Sought treatment from one source had a negative sign and was statistically significant at 99% level of confidence.

The interpretation of the results is easier with the odds ratio as they provide a better meaning. Using the place of treatment as an example, it is thus: for a child that goes to a private provider, the odds of incurring OOP expenditure are 13.4 times higher than the odds for a child that goes to a public facility. In the same way, a child whose mother is of higher school level education, the odds of incurring OOP expenditure are 4.3 times higher than the odds of a child whose mother has no education. The odds of incurring OOP expenditure is 2.6 times higher for individuals in the richest socio-economic category compared to those in the poorest.

The findings suggest that children in the districts of Kalangala, Masaka, Mpigi, Rakai, Lyantonde, Sembabule and Wakiso are less likely to incur OOP expenditure compared to the rest of the country – the central 1. In addition, those that seek health care from one source are less likely to have incurred OOP expenditure – with odds of one to 10 compared to multiple sources. Finally, disruption of normal activities is used as a proxy for severity of the illness and finds that when the illness is severe, OOP is likely to be incurred.

#### Levels and patterns of out-of-pocket expenditure

Levels and patterns of OOP expenditure are explored with a focus on how much is spent on average and overall, and on each of the components – consultation, medicine, transport, and hospitalization (Table 6). The average OOP expenditure for each child who incurred hospitalization expenses was US$7.6. This implies that on average, hospitalization was the most expensive. Similarly, the US$2.3 for medicines is the average for a child spent on medicines. Expected patterns emerge: the urban incur more than twice the amount compared to their rural counterparts, and the richest 20% incur more than three times the amount of the poorest 20%.

**Table 6:** Details of average OOP expenditure (US dollars) for given categories of the population on different expenditure items

**Background characteristics**	**Total**	**Consultation**	**Medicine**	**Transport**	**Hospitalization**
Residence					
Urban	7.26	4.85	4.35	1.65	7.05
Rural	3.39	2.85	2.06	2.35	7.69
Sub-region					
Central 1	4.57	2.14	3.88	1.91	17.24
Central 2	4.73	4.28	3.07	2.15	9.94
Kampala	5.06	2.96	5.04	1.10	8.03
East central	5.90	4.09	2.86	1.61	4.79
Mid-eastern	4.91	4.24	2.09	2.89	5.86
North-east	2.60	2.42	2.26	5.47	0.49
Mid-northern	1.58	1.14	0.98	2.79	3.13
West Nile	1.68	1.81	1.02	1.22	3.40
Mid-western	5.24	2.02	3.01	2.98	23.46
South-western	7.05	5.69	4.44	1.94	13.46
Mother's education level					
No education	3.92	2.85	2.56	2.13	4.89
Primary	3.17	2.83	1.86	2.33	10.02
Secondary	6.65	5.03	4.07	2.30	7.04
Higher	4.12	2.73	2.27	0.93	-
Wealth index					
Poorest	2.42	2.33	1.53	2.73	4.33
Poorer	3.03	2.80	1.68	2.82	3.70
Middle	3.59	2.98	2.29	1.61	11.76
Richer	4.17	3.46	2.28	2.14	8.19
Richest	7.87	4.63	5.02	1.91	23.92
Total	3.87	3.28	2.34	2.20	7.64

There are other unexpected patterns. In Kampala, the capital city, it would be expected that patients incur more OOP expenditure due to higher income levels. Nevertheless, Kampala is not the highest, and instead the districts of the west and east central are higher. Controlling for other factors, and using the districts of Kalangala, Masaka, Mpigi, Rakai, Lyantonde, Sembabule, and Wakiso as the base, this difference is not statistically significant.

Table 7 summarizes the Log-lin model regression estimates, i.e. slope coefficients and t-statistics. Once again, asterisks * and ** are used to indicate whether a slope coefficient is statistically significant at either 5% or 1%.

**Table 7:** Log-linmodel estimates for the natural log of OOP expenditure for children under five with malaria in Uganda

**ln(out-of-pocket)**	**Coef**	**Std. Err**	**t-stat**	**P>t**	
Wealth Index(Poorest)					
Poorer	0.20	0.12	1.69	0.091	
Middle	0.03	0.13	0.26	0.798	
Richer	0.10	0.14	0.75	0.453	
Richest	0.43	0.17	2.53	0.011	*
Rural	-0.28	0.19	-1.48	0.140	
Place first sought treatment (Public facility)^$^					
Private	-0.10	0.09	-1.17	0.243	
Traditional healer	-1.73	0.33	-5.20	0.000	**
Dont Know	-0.06	0.50	-0.11	0.909	
Sought treatment from one source	-0.56	0.13	-4.42	0.000	**
Highest educational level (no education)					
Primary	-0.26	0.10	-2.66	0.008	*
Secondary	0.19	0.15	1.25	0.213	
Higher	-0.05	0.33	-0.14	0.885	
Sub-region (Central 1)					
central 2	-0.13	0.20	-0.64	0.522	
Kampala	-0.24	0.32	-0.74	0.458	
east central	-0.39	0.21	-1.86	0.063	
mid eastern	-0.22	0.21	-1.04	0.299	
north east	-0.56	0.21	-2.68	0.007	*
mid northern	-1.02	0.20	-4.98	0.000	**
west nile	-0.96	0.21	-4.64	0.000	**
mid western	0.25	0.22	1.13	0.261	
south western	0.31	0.24	1.31	0.192	
Normal duties disrupted	0.49	0.09	5.70	0.000	**
Constant	9.44	0.48	19.73	0.000	
Number of observations				936	
Adj R-squared				0.21	
F(22, 913)				12.6	

** Statistically significant at 1% level.

*Statistically significant at 5% level.

In Log-lin model the coefficients for Traditional healer, Sought treatment from one source, Primary educational level, North East, Mid Northern and West Nile variables had a negative sign and were statistically significant at 95% level of confidence. Those who sought care from a traditional healer for malaria treatment incurred less OOP than those who went to public health facilities. Districts of north-east, mid-north and west Nile incur statistically significant less OOP expenditure than the base districts.

The slope coefficients for richest wealth quintile and Normal duties disrupted had a positive sign and were statistically significant at 95% and 99% level of confidence respectively. The richest families incurred higher OOP compared to the poorest segment.

## Discussion

The results are comparable to similar studies. Survey reports in Uganda show that malaria is the top cause of morbidity in the population [15]. Data collected through routine administration at heath units through the Health Management Information System also show malaria as a leading cause for outpatient attendance [5]. Beyond this, it is also associated with maternal morbidity and mortality [5]. This study has shown that Kampala and south-west sub-regions reported fewer children experiencing fever in the two weeks prior to the survey. Reasons for this are not clear because ownership of at least one insecticide-treated net, one of the most effective prevention measures, is slightly lower than in some other regions. In addition, there were no significant differences in exposure to malaria messages [12].

The fact that a significant proportion of the population with malaria seek treatment is not new. What is counterintuitive is the fact that children in rural areas seek health care more than their urban counterparts. This could be due to the fact that urban dwellers are relatively more educated, with a higher income and better access to pharmacies and drug shops and thus the tendency to self-treat. A study done in Burkina Faso showed that with respect to malaria, perceived efficacy of self-treatment tended to be high and therefore self-treatment was most prevalent with malaria [16]. This is something that needs further investigation.

The finding that private providers are a preferred source of malaria treatment is also not new [17]. Drug shops are part of the private sector. There is concern with drug shops because lay people do dispense medicines, at times in inadequate dosages, which may partially account for the drug resistance being experienced in Uganda and in many parts of Africa [18]. Rutebemberwa *et al*., in their study on utilization of private providers by febrile children, caretakers used drug shops and private clinics because these providers were perceived to be near and because of the possibility of getting treatment on credit [17]. Indeed, the average distance to a health facility is shorter for the private than for the public in Uganda [19]. In the 2008 National Service Delivery Survey, the distance to the nearest health facility was 5.7 km for the public and 4.7 km for private. In some sub-regions, it is up to a 3-km difference. The relatively new strategy of using trained community medicine distributors to dispense anti-malarials to children to enable fast access to treatment may be a step in the right direction although coverage is still very low. The percentage of households that reported presence of a community health worker distributing malaria medicines was below 15% except for the north-east, mid-north and west Nile regions [12].

The fact that urban dwellers, richest 20% and children whose mothers have a higher level of education are more likely to incur OOP expenditure may be expected given the fact that these groups of people have relatively better ability to pay. In terms of regions and districts, the fact that the Karamoja region and districts of north-east, mid-north and west Nile are less likely to incur OOP expenditure again may be explained by the relatively lower capacity to pay given the fact that the percentage of the population living below the poverty level is higher (46%) than the national average (25%) [20]. The urban–rural difference is not statistically significant but the poor-rich differences are sustained. This means that the price differences between urban and rural are not really much – the rural and urban people seem to face the same health care prices.

The finding that majority of respondents still pay in the context where user fees were abolished is in part explained by the choice of private provider [17] and the fact that they purchase medicines [21]. OOP expenditure is certainly something that has to be factored in the policy discussion on malaria in Uganda. Several studies have shown that use of private providers is associated with higher OOP payments. Van Damme *et al*., in their study on OOP expenditure on health in Cambodia, found that those who sought care in the private sector exclusively spent close to three times more that those who went to public providers [22]. Similarly, Manzi *et al*. found that those who visited private non-governmental facilities paid about 30 times more than those seeking care at government facilities [23].

### Financial protection

OOP expenditure is still prevalent and private provider is the preferred choice and as such, increasing public provision may not be the sole answer. Money is required when your child gets sick irrespective of whether one spends it on consultation, medicine, transport or hospitalization. Irrespective of whether one is in the bottom 20% or top 20%, one is likely to seek health care.

This study has shown that with regard to OOP expenditure the difference in spending between poor and rich socio-economic categories is significant, while differences between urban and rural areas are not significant. Similar findings have been reported by Onwujekwe *et al*. [24]. Significant differences in expenditure between the poor and rich were also shown in a study from Ghana that examined malaria treatment costs at household level where the cost of malaria care was estimated at 1% of household income among the rich as opposed to 34% among the poor [25]. The fact that hospitalization is the most expensive has been documented elsewhere [5]. This is because illness requires much more than treatment of uncomplicated malaria, more expensive medicines and more staff time [26].

The existing efforts to improve public health care delivery are lauded and they should continue. As the results indicate in this analysis, those that fall through the cracks will always be picked by public services, especially the poor. However a serious re-think on providing financial protection, irrespective of whether it is based on contributions or transfers, needs to take into consideration the OOP expenditure [27].

### Pricing of medicine

The share of total OOP expenditure that goes to medicine is greater than other components, both in terms of amount and as a component. In practice this means that patients flock to a pharmacy, drug shop or an ordinary seller of medicine, buy the medicine and administer to a child. The exception would be in a public facility where patients do not need to pay for consultation but can buy medicine once prescribed. This has implications for medicine pricing. Several studies have shown expenditure on medicines being the highest component of OOP expenditure on health [24,28]. Social marketing of malaria medicine could complement public provision given the preference for private providers across several groups [29]. Certainly medicine is free in public health facilities, but the results show that a significantly higher share of children is taken to private providers.

In the interest of ensuring access for all, creative ways of making medicines available at affordable prices among the private providers has the potential to reduce the burden that results from OOP expenditure, especially on medicine. The current efforts of social marketing of anti-malarials is a welcome development which should be encouraged [29,30]. This should be implemented alongside regulating the private sector to ensure provision of quality services and fair pricing. An example of such an initiative is the Consortium for ACT Private Sector Subsidy that provides subsidized pre-packaged artemisinin-based combination therapy (ACT) sold in private health units. At the moment the ACT Coartem® is provided free in public health units and sold expensively at private health units. This initiative is piloted in the districts of Kaliro, Pallisa, Budaka, and Kamuli in eastern Uganda as a partnership between the Ministry of Health, Medicines for Malaria Ventures, the National Drug Authority, PACE, Surgipharm, IDA Solutions and Malaria Consortium. However, the private drug shops are excluded from this pilot [30] yet several people do purchase from these outlets especially in rural areas where private pharmacies may not exist.

## Conclusion

Efforts to improve access to malaria treatment should explicitly incorporate efforts to protect households from high OOP expenditure. The private sector is a key partner but there is need to ensure fair/affordable pricing. This calls for provision of subsidies to enable the private sector to reduce prices, and put in place regulatory mechanisms. The government-led community-based distribution of anti-malarials strategy should be scaled to enable fast and affordable access treatment for children aged under five.

## Abbreviations

ACT: Artemisinin combination therapy; MoH: Ministry of Health; UBOS: Uganda Bureau of Statistics; Ushs: Uganda shillings.

## Competing interests

The authors declare that they have no competing interests.

## Authors’ contributions

JNO and FM contributed to conceptualization of the study analysis, interpretation of results and drafting of the manuscript; APO and LM contributed to interpretation of results and drafting of the manuscript. JMK contributed to analysis, interpretation of results and drafting of the manuscript. All authors read and approved the final manuscript.
